# Autism Spectrum Disorder Detection by Hybrid Convolutional Recurrent Neural Networks from Structural and Resting State Functional MRI Images

**DOI:** 10.1155/2023/4136087

**Published:** 2023-12-20

**Authors:** Emel Koc, Habil Kalkan, Semih Bilgen

**Affiliations:** ^1^Istanbul Okan University, Istanbul, Türkiye; ^2^Gebze Technical University, Darıca, Türkiye

## Abstract

This study aims to increase the accuracy of autism spectrum disorder (ASD) diagnosis based on cognitive and behavioral phenotypes through multiple neuroimaging modalities. We apply machine learning (ML) algorithms to classify ASD patients and healthy control (HC) participants using structural magnetic resonance imaging (s-MRI) together with resting state functional MRI (rs-f-MRI and f-MRI) data from the large multisite data repository ABIDE (autism brain imaging data exchange) and identify important brain connectivity features. The 2D f-MRI images were converted into 3D s-MRI images, and datasets were preprocessed using the Montreal Neurological Institute (MNI) atlas. The data were then denoised to remove any confounding factors. We show, by using three fusion strategies such as early fusion, late fusion, and cross fusion, that, in this implementation, hybrid convolutional recurrent neural networks achieve better performance in comparison to either convolutional neural networks (CNNs) or recurrent neural networks (RNNs). The proposed model classifies subjects as autistic or not according to how functional and anatomical connectivity metrics provide an overall diagnosis based on the autism diagnostic observation schedule (ADOS) standard. Our hybrid network achieved an accuracy of 96% by fusing s-MRI and f-MRI together, which outperforms the methods used in previous studies.

## 1. Introduction

Millions of neurons are responsible for coordinating each part of the human body and brain. When brain networks are incorrectly connected to coordinate activities, certain disorders in the human body arise [1, 2]. Some of the most common neurodevelopmental disorders are autism spectrum disorder (ASD) [3], schizophrenia [4], attention deficit hyperactivity disorder (ADHD) [5], epilepsy [6], Parkinson's disease [7], obsessive-compulsive disorder [8], and bipolar disorder (BD) [9].

ASD refers to a range of neurodevelopmental disorders with behavioral and cognitive impairments that place a huge burden on patients, families, and society. Identifying ASD patients directly in comparison to healthy controls is important for early detection and intervention. ASD's exact cause is still unknown [10]. Due to lack of knowledge of neuropathology, symptom-based diagnosis often results in poor treatment.

Early accurate diagnosis of ASD is pivotal to develop specialized interventions [11]. Due to its complex nature and highly heterogeneous symptoms, the diagnosis of ASD is very challenging [12].

Neuroimaging is an attractive noninvasive modality to cross the gap between environment, genes, and cognitive and behavioral phenotypes in ASD. Several studies in neuroimaging have used different techniques such as structural and functional magnetic resonance imaging (MRI) [12–17]. Similar studies have contributed to our understanding of brain changes in ASD subjects on structural and functional connectivity levels. Functional connectivity has been used to presage early autism diagnosis and restrict correlations within specific neural circuits across blood oxygenated level-dependent (BOLD) signals at different brain regions [18].

A number of studies have aimed to diagnose ASD based on structural magnetic resonance imaging (s-MRI) and functional magnetic resonance imaging (f-MRI) data [1]. In an earlier study, McKeown et al. anatomized f-MRI data into spatial components by blind separation [19]. Later, Uddin et al. presented a model using logistic regression classifier and independent component analyses in order to differentiate between diseased and health patient groups [20]. S-MRI data delineate the structural properties of the brain and have received attention from researchers [21–26]. Another study proposed a new model for distinguishing between ASD positive and negative individuals grounded on the features of s-MRI and f-MRI data using histogram of oriented gradients [27].

The goal of the present study is to formulate an effective machine learning (ML) architecture to enhance the effectiveness of ASD diagnosis. We aim to classify ASD patients and HC participants using s-MRI in conjunction with rs-f-MRI data from a large multisite data repository, namely, ABIDE (autism brain imaging data exchange). The dataset is phenotypically rich and consists of different modalities from an important clinical population. We also aim to identify significant brain connectivity features via functional connectivity classification of ASD patients and HC participants. We apply deep learning to identify ASD patients, grounded on the patient's brain blood oxygen level-dependent (BOLD) activation patterns. Multimodality fusion on s-MRI and f-MRI improves classification performance over the existing methods in our implementation. The proposed multimodality hybrid method achieves state of the art accuracy of 96% in distinguishing ASD from HC individuals. We benefited from the combination of convolutional neural networks (CNNs), which has strong modeling, and feature extraction power and recurrent neural networks (RNNs), which fused and ordered time series data. Furthermore, there are also privileges of the dataset and the atlas used for preprocessing.

## 2. Materials and Methods

### 2.1. Data Description

In the present study, both T1 weighted structural MRI and T2 weighted functional MRI data are obtained from image and data archive powered by laboratory of neuro imaging (LONI) [28] from ABIDE [29]. All data were used under the direction and approval of the respective institutions' ethics boards. ABIDE is based on a collaboration of 17 international imaging sites that have aggregated and are openly sharing neuroimaging data from 539 individuals suffering from ASD and 573 typical HC [30] in the neuroimaging informatics technology initiative (NIfTI) format. The data collected from these 1112 subjects consist of structural and resting state functional MRI data along with an extensive array of phenotypic information. All subjects have been selected by evaluating phenotypic information like age, gender, and intelligence. It is known that the scanning infrastructure in each imaging site used different parameters such as repetition time (TR), echo time (TE), number of voxels, number of volumes, openness or closeness of the eyes, and protocols for the data.

Fivefold cross validation strategy was used to evaluate the performance. In detail, each source was split into five subsets with an approximately equal number of subjects. We used four subsets of the data for training and the other for validation to select the model each time. Then, we conducted the adaptation process on time series cross validation. The augmented validation data were used during adaptation process.

In this study, we used the statistical parametric mapping (SPM) software version 12 (SPM12) built in MATLAB and computation, display, and analysis of connectivity (CONN) toolbox. SPM integrated toolbox was developed [31] as an extension to SPM for incorporating morphometric voxel-based (VBM), seed-based (SBM), or region of interest (ROI)-based neuroimaging methods.

F-MRI is a noninvasive technique to assess brain functions by using signal changes [14]. A group of small cubic elements referred as voxels represent the brain volume of f-MRI data. F-MRI consists of time series data extracted from each voxel by keeping track of its activity over time. The time series represent the signal measured at each voxel. Rs-f-MRI is used for analyzing brain disorders implementing f-MRI techniques while the subject is in a resting state. The major approach explored for discriminating between typically and autistic developed brains was shape and volumetric based analysis of s-MRI. S-MRI is generally classified as an anatomical study consisting of two categories of features, namely, shape features and volumetric features.

The heterogeneity of disorders of autistic individuals has increased the need for personalized approaches to analyze and prognosticate both functionally and anatomically for each autistic subject. Hence, in the present study, we combined s-MRI and f-MRI data with the aim of achieving better diagnostic accuracy and suggesting optimum treatment plan for every autistic subject. We analyze our results to ascertain that they fit better with autism diagnostic observation schedule (ADOS). Correlation is analyzed among all subjects for trait score differences and ADOS total scores to extract features of autism severity.

### 2.2. Data Preprocessing

**Figure 1:**
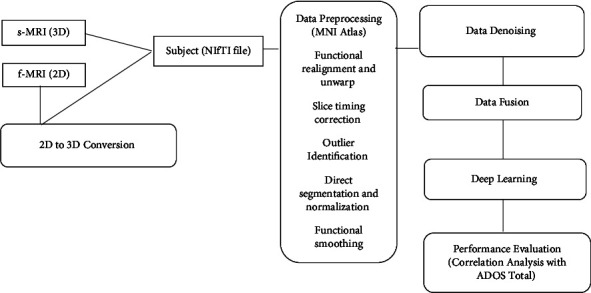
Overall pipeline of the proposed approach.

Neuroimages display thousands of cortical and subcortical areas, providing information on structures and functions. Brain atlases are used to divide brain images into a limited number of regions of interest (ROI) in order to overcome complexity [32].  Figure 1 depicts the overall pipeline of the approach we propose. For each modality, data preprocessing is necessary in order to avoid the risk of scanner bias and the effect of heterogeneity of protocols. In addition, the steps of denoising, fusion, and analysis to evaluate hybrid deep learning methods and correlation with ADOS total score are explained in the following sections.

First, in order to convert 2D f-MRI to 3D s-MRI, we used ROI percolation Harvard-Oxford atlas. Then, our preprocessing pipeline consisted of functional realignment and unwarp; slice-timing correction; outlier identification; direct segmentation and normalization; and functional smoothing within Montreal Neurological Institute (MNI) atlas. There are many studies using various atlases such as Harvard-Oxford (HO), Craddock 200 (CC200), Craddock 400 (CC400), Automated Anatomical Labeling (AAL), Eickhoff Zilles (EZ), Talaraich and Tournoux (TT), and Dosenbach 160 [1]. For the context of the present study, we downloaded the time series for the brain areas specified in MNI standard brain atlas [33]. In our literature review, we have realized that the MNI atlas has rarely been used with the large volume and different modality of ABIDE dataset. It is included in different neuroimaging analysis packages, including the statistical parametric mapping package (SPM). We have selected MNI atlas in order to perform comparisons across subjects and studies, particularly of subcortical data, which is accurately aligned by nonlinear volume registration in comparison to cortical data. In addition to that MNI atlas overcomes the neuroimage differences in shape, size, and relative orientation. The advantage of MNI atlas is that it focuses on disorders and artifacts on neuroimaging data used to analyze its functional and structural connectivity from the top portion of the brain to the bottom portion of the cerebellum [34].

Preprocessing is a significant step to remove the effects of different scanners, artifacts, or partial volume effects and the variability between subjects that may stem from data acquisition. In order to reduce execution time and achieve better accuracy, preprocessing of neuroimages generally consists in performing a fixed set of operations on the data. We used the CONN [35] functional connectivity toolbox that works with MATLAB/SPM. In order to reduce physiological and other noise sources, additional removal of movement and temporal covariates, temporal filtering and windowing of the residual BOLD contrast signal, first level estimation of multiple standard f-MRI and s-MRI measures, and second-level random-effect analysis, CONN provides a method as well as component based noise correction. Although global signal regression could also have been considered, the component based noise reduction method allows for interpretation of inverse correlations because there is no global regression signal in our implementation. The toolbox implements f-MRI and s-MRI measures, such as estimation of seed-to-voxel and ROI-to-ROI functional correlations, as well as semipartial correlation and bivariate/multivariate regression analysis for multiple ROI sources, graph theoretical analysis, and novel voxel-to-voxel analysis of functional connectivity.

In the course of functional realignment and unwarp, all neuroimages that belong to a subject are oriented in reference to the first image of the time series of that subject. The purpose of slice-timing correction is to set the time series of the voxel so that all the voxels in each image have a common reference time. Outlier identification scans are identified based on the observed global BOLD signal and the amount of subject motion. The change in the global BOLD signal at any time is calculated as the change in the average BOLD signal within SPM's global mean mask scaled to standard deviation units. In addition, we employ the relative probability densities of gray matter (GM), white matter (WM), and cerebrospinal fluid (CSF) in MNI space as inputs to the hybrid method. Therefore, direct segmentation provides segmentation into GM, WM, and CSF tissue classes. Also, direct normalization iteratively performs tissue classification from intensity values from functional and structural reference images and estimates nonlinear spatial transformations that approximate posterior and anterior tissue probabilities until convergence. Finally, data are smoothed in order to clean images of nonbrain artifacts from the series of voxels. This consists in averaging the neighbor voxel signals, as blood supply and its functions are usually close among neighboring brain voxels. Without disturbing the BOLD signal, temporal filtering eliminates redundant components from time series of voxels [36, 37].

### 2.3. Data Denoising

Using neuroimages in order to diagnose ASD is challenging due to the noise redounded from the image recording process. Consequently, there are many filtering approaches such as NLM filters, wavelet based filters, and band-pass filters, to extract the noise [38]. In this study, we prefer band-pass filtering for denoising the pipeline to reduce unwanted phase shifts.

MATLAB signal processing toolbox is particularly useful to filter signals with filter design parameters such as filter type, filter order, and attenuation. It combines two steps that use linear regression of potential artifacts in the BOLD signal and temporal band-pass filtering. BOLD signals are forecasted and removed separately for each voxel and for each subject due to factors identified as potential confounding effects. Working with this filtering, we resample all data to ensure equally spaced points for comparison into subjects. To that end, we use MATLAB function resample, which applies an antialiasing band-pass filter to the time series and compensates for the delay introduced by the filter. This function resamples the input sequence, the raw head motion in our case [39].

Inhomogeneity correction is applied to increase accuracy of artifacts in images created by nonhomogeneous brain tissues. Various techniques such as histogram matching are available for normalizing the volume of images [38].

While minimizing the effects of noise sources such as head movement and physiological variations, temporal frequencies below 0.008 Hz or above 0.09 Hz are removed from the BOLD signal using a band-pass filter [40].

**Figure 2:**
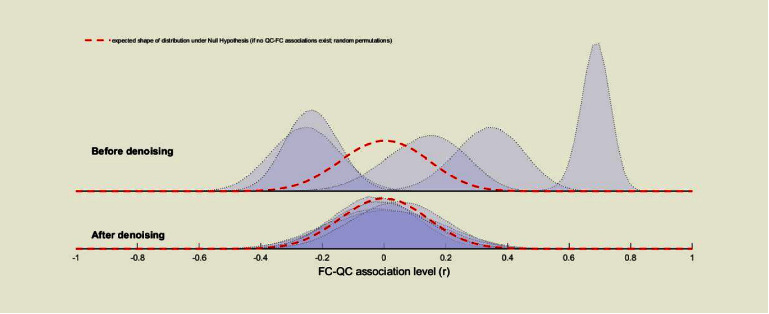
A sample of denoising output.

Figure 2 shows a sample of denoising output obtained from our dataset. Functional connectivity (FC) measures can be best classified by estimating the distribution of FC values between randomly selected pairs of points within the brain before and after denoising in order to minimize the effect of artifactual factors. After preprocessing pipeline but before denoising considering the BOLD signal, FC distributions show large intersession, intersubject variability with degrees of positive biases including large scale physiological, and subject motion effects. After denoising, FC measures orient approximately centered in the positive side with considerably reduced intersession and intersubject variability.

### 2.4. Classification Methods

**Figure 3:**
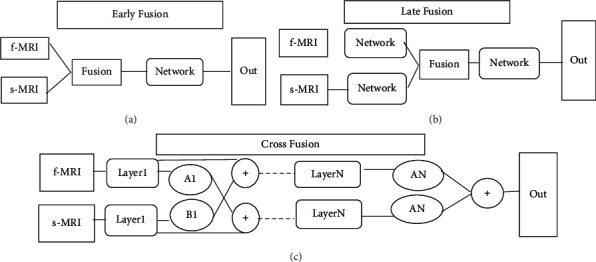
Framework of the fusion strategies. (a) Early fusion, (b) late fusion, and (c) cross fusion.

Investigating another line of research [41], the newly proposed cross fusion fully convolutional neural network (FCN) performed best among the multimodality and fusion networks. Based on that finding, three alternative fusion strategies were considered in the present work: early, late, and cross fusion, as shown in Figure 3.

**Figure 4:**
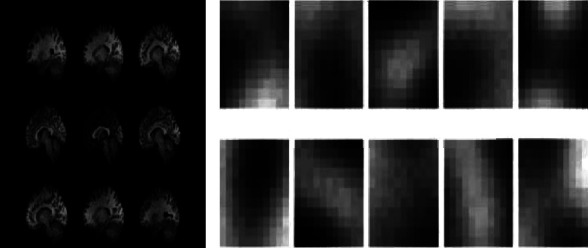
FCM images output from cross fusion with *α* = 0.05.

For early fusion (Figure 3(a)), the preprocessed f-MRI and s-MRI neuro images are combined for each subject thus producing a tensor. This input tensor is processed using the model network. For late fusion (Figure 3(b)), parallel streams process the f-MRI and s-MRI images independently before being fed into the model network. The output is fed through the neural network that carries out information fusion. For cross fusion (Figure 3(c)) which we propose, there are two processing branches connected by trainable scalar cross connections. The purpose of the process is to provide the functional connectivity matrix (FCM) information with cross-trainable fusion parameters rather than limiting the features to a single plane. The difference between cross fusion and studies in the related literature is the usage of hyper parameters. To overcome dimensional differences of feature matrices that belong to different neuro images during the pairwise comparison, training is carried out with a selected value of the parameter *α* (Figure 4). It was observed through trial runs that higher *α* value required almost prohibitive processing times and lower values resulted in unacceptably blurred images. Thus, *α* = 0.05 was selected to provide acceptable image quality with available processing power. During training, the parameter is automatically adjusted to integrate two different information modalities f-MRI and s-MRI.

With the scalar crosslinks formed with A1 (*α*) and B1 (*α*) in layer 1, *N* ∈ {0, 0.01, 0.02,…, 0.09, 1} probabilities of each layer are calculated within the cross fusion. *α* controls the gradient range. To further demonstrate the effects of *α* on fusion results, we have selected threshold of *α* = 0.05. The FCM image (Figure 4) shows areas where gray matter, white matter, and CSF features are clustered.

Figure 4 left side shows a sample of preprocessed cross-sectional volumes and right side shows their corresponding feature maps. In addition, each subimage corresponds to a single filter. The convolutional filters are sensitive to features of the preprocessed cross-sectional volumes of the patients with a diagnosis of ASD.

To tackle the high dimensionality of the acquired features, we selected tissue kind as a feature. In the literature, several novel CNN or RNN models were constructed to create different features with different configuration parameters. By taking inspiration from them, we selected only different tissue area-related features. The maps in Figure 4 are shown with the descriptive information of the clusters obtained at the selected significance level.

After data preprocessing and denoising, the first stage of our framework consists of a CNN and an RNN in a hybrid form. The main idea of these networks is to use a convolutional layer. Both networks are used to detect spatial dependencies in data within the help of the convolution layer [42]. In order to analyze multidimensional time series, CNN and RNN are useful [43]. The advantage of this model lies in the possibility of using a pretrained model.

**Figure 5:**
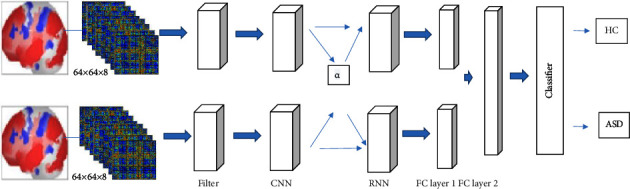
Overall block diagram of a CNN-RNN used for ASD detection.

CNN has three introductory layers referred as fully connected convolution layer, pooling layer, and the final convolution layer. First, the input signal is directly connected to the convolution layer and a kernel is used for convolution operation. In addition, operation results are created as a feature map for the next layer. Between two layers of convolution is a layer of pooling. In order to reduce the size of feature mat, the pooling layer is used. Otherwise, inside the same hidden layer, RNN sends feedback signals to the other neurons within the related layer (Figure 5). The output of the CNN layer was created by selecting *α* parameter of 0.05 and given as input to the RNN layer. Then, the feature vector is formed with the RNN output. In the fully connected layer, performance evaluation was made first separately and then by combining subject together with concatenation of data. At the last stage, classifier and output process takes place and the model result is parsed as ASD and HC.

We have used Matlab/SPM based cross platform software on Windows environment on an Intel Core i7 processor, a clock frequency of 3 GHz, 32 GB RAM, 500 GB Solid State Drive (SSD) computer. Training our network took a little over 2 hours per epoch and around 2 days and a half for the fully trained hybrid convolutional recurrent neural networks. Number of iterations is the number of passes, each pass processing data that belong to all subjects. Our method takes on average 2-3 minutes to segment the data of a single subject from the ABIDE dataset (nearly two days for all 1112 subjects). In high performance computing environments, CONN can distribute our processing and analyses in parallel across multiple nodes. This can result in a very significant reduction in processing time.

For each pair of subjects, Pearson's correlation coefficients have been used with ADOS report. It is significant to have multiplicity adjustments to control the false discovery rate (FDR) for the test. In this study, we have applied the FDR with the threshold of 0.1 for correlation analysis [44].

## 3. Results

### 3.1. Summary Statistics

There is no public dataset available consisting of data from different modalities such as electroencephalography (EEG), diffusion tensor imaging (DTI), MRI, and f-MRI (resting state and task based), that belong to the same individuals. Furthermore, there is a lack of ASD subsyndromes data such as Asperger's syndrome (AS) [45] and pervasive developmental disorder, not otherwise specified (PDD-NOS) [46], and distribution rates according to number of samples by gender are also low. For future studies, availability of datasets that provide different modalities will help researchers to improve ASD detection accuracy using ML and deep learning methods.

We observed that the combination of ML classifiers with other clinical features of ASD improved the accuracy of ASD diagnosis. The current sample size identifies relatively relevant brain regions at high risk for ASD, suggesting that this method can be extended to large and more heterogeneous ASD populations. Using s-MRI and f-MRI modalities in conjunction, we have shown that a higher level of diagnosis accuracy can be achieved.

For each subject, local diagnosis accuracy for both s-MRI and f-MRI feature matrices is calculated.  Table 1 shows the accuracy, sensitivity, and specificity obtained for s-MRI and f-MRI when using all features. Accuracy measures the proportion of correct predictions made by the model. It is defined as the ratio of the number of correct predictions to the total number of predictions made. Sensitivity measures the proportion of actual positives that are correctly identified as positive by the model. It is defined as the ratio of the number of true positives to the total number of actual positives. And also, specificity measures the proportion of actual negatives that are correctly identified as negative by the model. It is defined as the ratio of the number of true negatives to the total number of actual negatives.  Table 2 shows the accuracy achieved by different fusion (early, late, and cross) strategies. As can be seen, cross fusion with ADOS yielded the highest accuracy among the other fusions. We do not prefer late and cross fusion processes without ADOS because the score obtained with ADOS is consistently higher than that obtained without ADOS. Our results show that the hybrid model, achieving classification performances of 96.02%, 92.83%, and 85.70% for the accuracy, sensitivity, and specificity, respectively, is significantly superior to the single CNN and RNN models.

Our hybrid algorithm provides high accuracy and specificity when s-MRI and f-MRI are analyzed together. Our model also fuses the s-MRI and f-MRI datasets, which provides an accuracy of 96.02% accuracy, higher than alternatives.

We have investigated the effects of different s-MRI and f-MRI parameters on the machine learning algorithm. Proposed diagnosis may get better via both modalities, and we have observed that the addition of s-MRI and f-MRI parameters in features specific for ASD classification gives a higher significant Pearson correlation at *P* = 0.001 than benchmark data with ADOS total score. Thus, the current data suggest that the approach of a localized diagnosis with fusion of different modality datasets, fusion strategies, and correlation to ADOS will greatly improve accuracy, sensitivity, and specificity.

In Table 3, we compare individual CNN, RNN, hybrid CNN-RNN, and other recent machine learning methods with similar studies, albeit on different datasets and different diseases, based on the usage of neuroimaging data, in terms of accuracy. Studies using CNN, only RNN, their combination, and other methods are shown. A study reports a CNN study with a very high accuracy of 100 percent for Alzheimer disease Hossesini-asl et al. [47], another one presents a two-dimensional CNN with the high accuracy of 90.29 percent for hyperactivity disease [71], and other one achieves an accuracy of 98.8 percent for Parkinson's disease [58]. Among the studies that utilized the Parkinson's disease dataset, the study achieved an accuracy of 82.89 percent using both CNN and RNN, which is a hybrid method [65]. Researchers show the usefulness of ML techniques to identify and predict generalized disease. Application of ML technique in EEG of patients with epilepsy is very recent and is emerging with promising results within balanced accuracy of 98.13% [70]. In addition, in Table 4, we compare different ASD studies in which machine learning methods have been applied on different sets of neuroimaging data, different modalities, and different ML methods. Another inspiring publication showed that the computer-aided diagnosis system was able to accurately distinguish between individuals with ASD and controls, achieving an accuracy rate of 87.1% [15]. Yet another more recent work by the same author [18] demonstrates the potential of using dynamic functional connectivity analysis to identify brain regions associated with specific symptoms of ASD with 47 subjects which is lower than we are. By identifying these regions, the author aims to contribute the development of more targeted and personalized interventions for individuals with ASD. Many studies in the literature have focused on group level differences between individuals with ASD and typically developing controls. While these studies have identified some brain regions consistently associated with ASD, they do not account for the variability in brain structure and function that exist within the ASD population. Another difference between our study and some related studies is the use of a combination of s-MRI and f-MRI data. The combination of these two types of data allows for a more comprehensive analysis of brain structure and function, which may improve the accuracy of ASD diagnosis. Researchers have developed several approaches for seizure detection using ML classifiers and statistical features [88, 94]. A recent publication [84] demonstrates substantial difference in the efficiency and accuracy of various biomarkers used for ASD diagnosis. The difference in the performance of various biomarkers is due to heterogeneity of ASD. Our fusion of f-MRI and s-MRI data has improved the accuracy of existing autism detection systems by combining two modalities. Some studies in the literature have investigated special biomarkers consisting of biological molecules used for biomedical imaging and neuromodulation. In the present study, we did not investigate biomarkers but rather focused on algorithmic enhancement of accuracy. In addition, we combined CSF with WM and GM. Our machine learning methodology and fusion strategies are different from that applied by Jamwal et al. achieving higher accuracy via a novel neural network structure.

## 4. Conclusion

In general, it is difficult to generalize the findings of studies utilizing a small selection of samples. In addition, many studies in related areas focus on different age populations, thus limiting generalizability. Studies in the literature that focus on gender differences also inevitably reduce sample sizes, leading to reduced statistical confidence. An important challenge of neuroimaging datasets is the unavailability of different modalities. By using the ABIDE dataset, we were able to overcome these challenges, through utilizing s-MRI and f-MRI data together for a large number of subjects. Clinical studies have shown that using multimodality techniques play a significant role in increasing the accuracy of ASD diagnosis [97]. Our contribution can be summarized as implementing different modality fusion with higher accuracy and correlation with ADOS within a hybrid method consisting of CNN and RNN.

Future direction in the path towards more effective ASD diagnosis and treatment is expected to further exploit the potential of hybrid ML algorithms for classification. Local analysis of the brain regions is expected to enable clinicians to deliver personalized treatments to autistic individuals. And also, our cross fusion infrastructure will be provide region based analysis of the brain, which we believe that it can allocate subjects on the autism spectrum and help clinicians deliver personalized treatments to individuals with autism. Another possibility that has emerged with our approach is the integration of further imaging modalities such as DTI and EEG data to diagnostic studies based on neuroimaging, in order to obtain a higher number of features and using biomarkers to improve classification accuracy. In addition, subcategorization of autistic disorders such as Asperger and PDD-NOS via multimode neuroimaging may become possible using the proposed hybrid ML approach.

## Figures and Tables

**Table 1:** Performance comparison among various ML methods applied on the ABIDE dataset.

	CNN (%)	RNN (%)	Hybrid (CNN + RNN) without ADOS (%)	Hybrid (CNN + RNN) with ADOS (%)
Accuracy	67.65	60.30	92.89	96.02
Sensitivity	57.02	62.30	85.63	92.83
Specificity	78.33	58.90	96.81	85.70

**Table 2:** Accuracies achieved with different fusion strategies.

Hybrid (CNN + RNN)	With ADOS (%)	Without ADOS (%)
Early fusion	95.44	92.89
Cross fusion	96.02	—
Late fusion	95.24	—

**Table 3:** Recent studies in the literature non-ASD.

Studies	Method	Year	Modality	Data set	Accuracy (%)
Hosseini-Asl et al. [47]	3D CNN	2018	3D s-MRI	Alzheimer	100
Wang et al. [48]	CNN	2018	s-MRI	Alzheimer	97.65
Duc et al. [49]	3D CNN	2020	f-MRI	Alzheimer	85.27
Spasov et al. [50]	3D CNN	2018	s-MRI	Alzheimer	99
Liu et al. [51]	ResNet/3D DenseNet	2020	s-MRI	Alzheimer	88.9
Farooq et al. [52]	GoogleNet/ResNet	2017	s-MRI	Alzheimer	98.88
Korolev et al. [53]	3D CNN	2017	3D s-MRI	Alzheimer	80
Senanayake et al. [54]	ResNet/DenseNet/GoogleNet	2018	3D MRI	Alzheimer	79
Zou et al. [55]	3D CNN	2017	rs-f-MRI	Hyperactivity	65.67
Zou et al. [56]	3D CNN	2017	f-MRI—s-MRI	Hyperactivity	69.15
Chen et al. [26]	3D CNN/2D CNN	2019	EEG	Hyperactivity	90.29
Campese et al. [57]	SVM, 2D CNN	2019	2D/3D s-MRI	Bipolar	86.30
Choi et al. [58]	3D CNN	2017	Computerized tomography	Parkinson	98.8
Wang et al. [21]	LSTM	2018	Array collection data	Alzheimer	99
Dakka et al. [59]	LSTM	2017	4D f-MRI	Schizophrenia	66.4
Kumar et al. [60]	RNN	2019	CT—MRI—PET	Alzheimer/Autism	91.9
Talathi [61]	GRU	2017	EEG	Epilepsy	99.6
Che et al. [62]	GRU	2017	Parkinson dataset	Parkinson	95.7
Yao et al. [63]	IndRNN	2019	EEG	Epilepsy	87
Zhao et al. [64]	GAN	2020	f-MRI	Mental	82.1
Shi et al. [65]	CNN/RNN	2019	Resting state EEG	Parkinson	82.89
Kim et al. [66]	SVM, logistic, k-NN, Naïve Bayes, random forest, AdaBoost, GBM, XGBoost	2023	PET validated EEG	Alzheimer	90.9
De Nedai et al. [67]	Unsupervised machine learning	2023	f-MRI	Obsessive-compulsive disorder	65.9
Hámori et al. [68]	SVM	2023	Event related potential (ERP)	Hyperactivity	78
Cha et al. [8]	Light GBM, XGBoost GBM	2023	Diffusion tensor imaging (DTI)	Obsessive-compulsive disorder	76.72
Belić et al. [69]	Sensor-based image	2023	k-NN	Parkinson	85.18
Escobar-Ipuz et al. [70]	EEG	2023	XGB, k-NN, decision tree, Naïve Bayes	Epilepsy	98.13

CNN: convolutional neural network; 3D: three-dimensional; 2D: two-dimensional; ResNet: residual networks; DenseNet: densely connected networks; MRI: magnetic resonance imaging; f-MRI: functional MRI; s-MRI: structural MRI; EEG: electroencephalography; SVM/SVC: support vector machine; CT: computed tomography; PET: positron emission tomography; RNN: recurrent neural network; LSTM: long-short term memory; GRU: gated recurrent unit; IndRNN: independent RNN; GAN: generative adversarial network; k-NN: k-Nearest Neighbors; GBM: gradient boosting machine; XGB: extreme gradient boosting.

**Table 4:** Recent studies in the literature with ASD.

Studies	Method	Year	Modality	Accuracy (%)
Zhao et al. [12]	SVM	2020	f-MRI	83
Mostafa et al. [72]	LDA	2019	f-MRI	77.70
Liu et al. [73]	DFC + MTFS	2020	rs-f-MRI	76.80
Wang et al. [74]	MLP + ensemble learning	2020	f-MRI	74.52
Sun et al. [75]	No superparameter FCN	2021	rs-f-MRI	71.74
Shi et al. [76]	Three-way decision model	2021	f-MRI	71.35
Grana and Silva [77]	SVC	2021	rs-f-MRI	71.10
Spera et al. [78]	L-SVM	2019	rs-f-MRI	71
Reiter et al. [79]	RF	2021	rs-f-MRI	67.81
Chaitra et al. [80]	RCE-SVM	2020	f-MRI	67.30
Brahim and Farrugia [81]	RBF-SVC	2020	rs-f-MRI	66.70
Sun et al. [82]	RBF + SVM	2021	f-MRI	59.10
Kazeminejad and Sotero [83]	NEG + MLP	2020	rs-f-MRI	58.70
Dekhil et al. [15]	Correlation analysis	2021	rs-f-MRI	95–100
Dekhil et al. [18]	k-NN, random forest	2019	s-MRI, f-MRI	81
Jamwal et al. [84]	DBN	2022	s-MRI, f-MRI	—
Traut et al. [85]	Logistic regression, SVC	2022	s-MRI, f-MRI	79
Dadi et al. [86]	K-means, wards algorithm, CanICA, DictLearn	2019	rs-f-MRI	86
Abraham et al. [87]	K-means, wards algorithm, ICA	2017	rs-f-MRI	67
Gogoi et al. [88]	VGG16, inception v3, ResNet50	2023	MRI	94
Han et al. [89]	Cross, supervised, LOSO, Fed_DA,	2023	rs-f-MRI, s-MRI	69.37
Manikantan and Jaganathan [90]	Graphical neural networks	2023	rs-f-MRI, s-MRI	69.45
Deng et al. [91]	GAN	2023	f-MRI	71
Dhinagar et al. [92]	Metalearning	2023	MRI	85.70
Artiles et al. [93]	Multiple linear regression	2023	rs-f-MRI	76.40
Quiang et al. [94]	Hierarchical FBN	2023	f-MRI	82.10
Jönemo et al. [95]	3D CNN	2023	rs-f-MRI	80
Kunda et al. [96]	MIDA, ridge classifier, logistic regression, SVM	2023	rs-f-MRI	73

SVM: support vector machine; LDA: linear discriminant analysis; DFC: dynamic functionally connected; MTFS: multitask feature selection; MLP: multilayer perceptron; FCN: functionally connected network; L-SVM: linear kernel support vector machine; RF: random forest; RCE: recursive cluster removal; RBF: radial basis function; NEG: negative correlation matrix; k-NN: k-nearest neighbors; DBN: deep belief network; CanICA: canonical independent component analysis; DictLearn: dictionary learning; ICA: independent component analysis; VGG: very deep convolutional networks; LOSO: leave one site out; Fed_DA: federated domain adaptation; GAN: adversarial generation network; FBN: functional brain networks; CNN: convolutional neural network; MIDA: maximum independence domain adaptation.

## Data Availability

The data used to support the findings of this study are available from the corresponding author upon request.

## References

[1] Khodatars M., Shoeibi A., Ghassemi N. (2021). Deep learning for neuroimaging-based diagnosis and rehabilitation of autism spectrum disorder: a review. https://arxiv.org/abs/2007.01285.

[2] Loh H. W., Ooi C. P., Barua P. D., Palmer E. E., Molinari F., Acharya U. (2022). Automated detection of ADHD: current trends and future perspective. *Computers in Biology and Medicine*.

[3] Yang X., Zhang N., Schrader P. (2022). A study of brain networks for autism spectrum disorder classification using resting state functional connectivity. *Machine Learning with Applications*.

[4] Sadeghi D., Shoeibi A., Ghassemi N. (2022). An overview of artificial intelligence techniques for diagnosis of Schizophrenia based on magnetic resonance imaging modalities. Methods, challenges and future works. *Computers in Biology and Medicine*.

[5] Bakhtyari M., Mirzaei S. (2022). ADHD detection using dynamic connectivity patterns of EEG data and ConvLSTM with attention framework. *Biomedical Signal Processing and Control*.

[6] Shoeibi A., Ghassemi N., Khodatars M., Jafari M., Moridian P., Alizadehsani R. (2021). Applications of epileptic seizures detection in neuroimaging modalities using deep learning techniques: methods, challenges and future works. https://arxiv.org/abs/2105.14278.

[7] Sahu L., Sharma R., Sahu I., Das M., Sahu B., Kumar R. (2022). Efficient detection of Parkinson’s disease using deep learning techniques over medical data. *Expert Systems*.

[8] Cha J., Kim B., Kim G. (2023). White matter diffusion estimates in obsessive-compulsive disorder across 1,653 individuals: machine learning findings from the enigma ocd working group. https://www.zora.uzh.ch/id/eprint/238344/.

[9] Highland D., Zhou G. (2022). A review of detection techniques for depression and bipolar disorder. *Smart Health*.

[10] Zaky E. A. (2017). Autism spectrum disorder (ASD); the past, the present, and the future. *Journal of Child and Adolescent Behavior*.

[11] Zwaigenbaum L., Bauman M. L., Stone W. L. (2015). Early identification of autism spectrum disorder: recommendations for practice and research. *Pediatrics*.

[12] Zhao F., Zhang H., Rekik I., An Z., Shen D. (2018). Diagnosis of autism spectrum disorders using multi-level high-order functional networks derived from resting-state functional MRI. *Frontiers in Human Neuroscience*.

[13] Anderson J. S., Nielsen J. A., Froehlich A. L. (2011). Functional connectivity magnetic resonance imaging classification of autism. *Brain*.

[14] Eslami T., Mirjalili V., Fong A., Laird A. R., Saeed F. (2019). ASD-DiagNet: a hybrid learning approach for detection of autism spectrum disorder using f-MRI data. *Frontiers in Neuroinformatics*.

[15] Dekhil O., Ali M., El-Nakieb Y. (2019). A personalized autism diagnosis cad system using a fusion of structural MRI and resting-state functional MRI data. *Frontiers in Psychiatry*.

[16] Heinsfeld A. S., Franco A. R., Craddock R. C., Buchweitz A., Meneguzzi F. (2018). Identification of autism spectrum disorder using deep learning and the abide dataset. *NeuroImage: Clinica*.

[17] Brown C. J., Hamarneh G. (2016). Machine learning on human connectome data from MRI. https://arxiv.org/abs/1611.08699.

[18] Dekhil O., Shalaby A., Soliman A. (2021). Identifying brain areas correlated with ADOS raw scores by studying altered dynamic functional connectivity patterns. *Medical Image Analysis*.

[19] McKeown M. J., Makeig S., Brown G. G. (1998). Analysis of f-MRI data by blind separation into independent spatial components. *Human Brain Mapping*.

[20] Uddin L. Q., Supekar K., Lynch C. J. (2013). Salience network-based classification and prediction of symptom severity in children with autism. *JAMA Psychiatry*.

[21] Wang J., Wei Q., Bai T. (2017). Electroconvulsive therapy selectively enhanced feedforward connectivity from fusiform face area to amygdala in major depressive disorder. *Social Cognitive and Affective Neuroscience*.

[22] Wang T., Qiu R. G., Yu M. (2018c). Predictive modeling of the progression of Alzheimer’s disease with recurrent neural networks. *Scientific Reports*.

[23] Wang J., Becker B., Wang L., Li H., Zhao X., Jiang T. (2019). Corresponding anatomical and coactivation architecture of the human precuneus showing similar connectivity patterns with macaques. *NeuroImage*.

[24] Wu H., Sun H., Wang C. (2017). Abnormalities in the structural covariance of emotion regulation networks in major depressive disorder. *Journal of Psychiatric Research*.

[25] Xu J., Wang J., Bai T. (2019). Electroconvulsive therapy induces cortical morphological alterations in major depressive disorder revealed with surface-based morphometry analysis. *International Journal of Neural Systems*.

[26] Chen R., Jiao Y., Herskovits E. H. (2011). Structural MRI in autism spectrum disorder. *Pediatric Research*.

[27] Ghiassian S., Greiner R., Jin P., Brown M. R. G. (2016). Using functional or structural magnetic resonance images and personal characteristic data to identify ADHD and autism. *PLoS One*.

[28] Craddock C., Benhajali Y., Chu C. (2013). The neuro bureau preprocessing initiative: open sharing of preprocessed neuroimaging data and derivatives. https://www.frontiersin.org/10.3389/conf.fninf.2013.09.00041/event_abstract.

[29] ABIDE (2011). Autism brain imaging data exchange. http://fcon_1000.projects.nitrc.org/indi/abide/.

[30] loni (2021). LONI Image and Data Archive. https://ida.loni.usc.edu/login.jsp?project=&page=HOME.

[31] Er F., Goularas D. (2021). Predicting the prognosis of MCI patients using longitudinal MRI data. *IEEE/ACM Transactions on Computational Biology and Bioinformatics*.

[32] Maldjian J. A., Laurienti P. J., Kraft R. A., Burdette J. H. (2003). An automated method for neuroanatomic and cytoarchitectonic atlas-based interrogation of f-MRI data sets. *NeuroImage*.

[33] Brett M. (2002). The MNI brain and the talairach atlas. https://imaging.mrc-cbu.cam.ac.uk/imaging/MniTalairach.

[34] Mandal P. K., Mahajan R., Dinov I. D. (2012). Structural brain atlases: design, rationale, and applications in normal and pathological cohorts. *Journal of Alzheimer’s Disease*.

[35] Conn (2020). Toolbox. https://web.conn-toolbox.org/.

[36] Jaber H. A., Aljobouri H. K., Çankaya I., Koçak O. M., Algin O. (2019). Preparing f-MRI data for postprocessing: conversion modalities, preprocessing pipeline, and parametric and nonparametric approaches. *IEEE Access*.

[37] Park B. Y., Byeon K., Park H. (2019). FuNP (fusion of neuroimaging preprocessing) pipelines: a fully automated preprocessing software for functional magnetic resonance imaging. *Frontiers in Neuroinformatics*.

[38] Manjon J. V. (2017). MRI preprocessing. *Imaging Biomarkers*.

[39] Caballero C., Mistry S., Vero J., Torres E. B. (2018). Characterization of noise signatures of involuntary head motion in the autism brain imaging data Exchange repository. *Frontiers in Integrative Neuroscience*.

[40] Hallquist M. N., Hwang K., Luna B. (2013). The nuisance of nuisance regression: spectral misspecification in a common approach to resting-state f-MRI preprocessing reintroduces noise and obscures functional connectivity. *NeuroImage*.

[41] Caltagirone L., Bellone M., Svensson L., Wahde M. (2018). *LIDAR-camera Fusion for Road Detection Using Fully Convolutional Neural Networks*.

[42] Wang J., Yang Y., Mao J., Huang Z., Huang C., Xu W. Cnn-rnn: a unified framework for multi-label image classification.

[43] Fan Y., Lu X., Li D., Liu Y. Video-based emotion recognition using CNN-RNN and C3D hybrid networks.

[44] Feliciano P., Zhou X., Astrovskaya I. (2019). Exome sequencing of 457 autism families recruited online provides evidence for autism risk genes. *Npj Genomic Medicine*.

[45] Attwood T. (2006). Asperger’s syndrome. *Tizard Learning Disability Review*.

[46] Towbin K. E. (2005). Pervasive developmental disorder not otherwise specified. *Handbook of autism and pervasive developmental disorders*.

[47] Hosseini-Asl E., Ghazal M., Mahmoud A., Aslantas A., Shalaby A. M., Casanova M. F. (2018). Alzheimer disease diagnostics by a 3D deeply supervised adaptable convolutional network. *Frontiers in Bioscience*.

[48] Wang J., Feng X., Wu J. (2018). Alterations of gray matter volume and white matter integrity in maternal deprivation monkeys. *Neuroscience*.

[49] Duc N. T., Ryu S., Qureshi M. N. I., Choi M., Lee K. H., Lee B. (2020). 3D-deep learning based automatic diagnosis of Alzheimer’s disease with joint MMSE prediction using resting-state f-MRI. *Neuroinformatics*.

[50] Spasov S. E., Passamonti L., Duggento A., Lio P., Toschi N. A. (2018). A multi-modal convolutional neural network framework for the prediction of alzheimer’s disease. *Annual International Conference of the IEEE Engineering in Medicine and Biology Society (EMBC)*.

[51] Liu M., Li F., Yan H., Wang K., Ma Y. (2020). A multi-model deep convolutional neural network for automatic hippocampus segmentation and classification in Alzheimer’s disease. *Neuroimage*.

[52] Farooq A., Anwar S., Awais M., Rehman S. A. Deep CNN based multi-class classification of alzheimer’s disease using MRI.

[53] Korolev S., Safiullin A., Belyaev M., Dodonova Y. Residual and plain convolutional neural networks for 3D brain MRI classification.

[54] Senanayake U., Sowmya A., Dawes L. Deep fusion pipeline for mild cognitive impairment diagnosis.

[55] Zou L., Zheng J., McKeown M. J. Deep learning based automatic diagnoses of attention deficit hyperactive disorder.

[56] Zou L., Zheng J., Miao C., Mckeown M. J., Wang Z. J. (2017). 3D CNN based automatic diagnosis of attention deficit hyperactivity disorder using functional and structural MRI. *IEEE Access*.

[57] Campese S., Lauriola I., Scarpazza C., Sartori G., Aiolli F., Oneto L., Navarin N., Sperduti A., Anguita D. (2019). Psychiatric disorders classification with 3D convolutional neural networks. *Recent Advances in Big Data and Deep Learning*.

[58] Choi H., Ha S., Im H. J., Paek S. H., Lee D. S. (2017). Refining diagnosis of Parkinson’s disease with deep learning-based interpretation of dopamine transporter imaging. *NeuroImage: Clinica*.

[59] Dakka J., Bashivan P., Gheiratmand M., Rish I., Jha S., Greiner R. (2017). Learning neural markers of schizophrenia disorder using recurrent neural networks. https://arxiv.org/abs/1712.00512.

[60] Kumar P. S. J., Yuan Y., Yung Y., Hu W., Pan M., Li X., Kumar P. S. J. (2019). Bi-directional recurrent neural networks in classifying dementia, Alzheimer’s disease and autism spectrum disorder. *The Art of Fixing Alzheimer’s Disease*.

[61] Talathi S. S. (2017). Deep recurrent neural networks for seizure detection and early seizure detection systems. https://arxiv.org/abs/1706.03283.

[62] Che C., Xiao C., Liang J., Jin B., Zho J., Wang F. An RNN architecture with dynamic temporal matching for personalized predictions of Parkinson’s disease.

[63] Yao X., Cheng Q., Zhang G. Q. (2019). A novel independent rnn approach to classification of seizures against non-seizures. https://arxiv.org/abs/1706.03283.

[64] Zhao F., Chen Z., Rekik I., Lee S. W., Shen D. (2020). Diagnosis of autism spectrum disorder using central moment features from low and high order dynamic resting state functional connectivity networks. *Frontiers in Neuroscience*.

[65] Shi X., Wang T., Wang L., Liu H., Yan N. (2019). Hybrid convolutional recurrent neural networks outperfom CNN and RNN in task-state EEG detection for Parkinson’s disease. *ResearchGate*.

[66] Kim N. H., Park U., Yang D. W., Choi S. H., Youn Y. C., Kang S. W. (2023). PET-validated EEG-machine learning algorithm predicts brain amyloid pathology in pre-dementia Alzheimer’s disease. *Scientific Reports*.

[67] De Nadai A. S., Fitzgerald K. D., Norman L. J. (2023). Defining brain-based OCD patient profiles using task-based fMRI and unsupervised machine learning. *Neuropsychopharmacology*.

[68] Hámori G., File B., Fiáth R. (2023). Adolescent ADHD and electrophysiological reward responsiveness: a machine learning approach to evaluate classification accuracy and prognosis. *Psychiatry Research*.

[69] Belić M., Radivojević Z., Bobić V., Kostić V., Đurić-Jovičić M. (2023). Quick computer aided differential diagnostics based on repetitive finger tapping in Parkinson’s disease and atypical parkinsonisms. *Heliyon*.

[70] Escobar-Ipuz F. A., Torres A. M., García-Jiménez M. A., Basar C., Cascón J., Mateo J. (2023). Prediction of patients with idiopathic generalized epilepsy from healthy controls using machine learning from scalp EEG recordings. *Brain Research*.

[71] Chen H., Song Y., Li X. (2019). Use of deep learning to detect personalized spatial-frequency abnormalities in EEGs of children with ADHD. *Journal of Neural Engineering*.

[72] Mostafa S., Tang L., Wu F. X. (2019). Diagnosis of autism spectrum disorder based on eıgenvalues of brain networks. *IEEE Access*.

[73] Liu J., Sheng Y., Lan W., Guo R., Wang Y., Wang J. (2020). Improved asd classification using dynamic functional connectivity and multi-task feature selection. *Pattern Recognition Letters*.

[74] Wang S. H., Phillips P., Sui Y., Liu B., Yang M., Cheng H. (2018). Classification of Alzheimer’s disease based on eight-layer convolutional neural network with leaky rectified linear unit and max pooling. *Journal of Medical Systems*.

[75] Sun L., Xue Y., Zhang Y., Qiao L., Zhang L., Liu M. (2021). Estimating sparse functional connectivity networks via hyperparameter-free learning model. *Artificial Intelligence in Medicine*.

[76] Shi C., Xin X., Zhang J. (2021). Domain adaptation using a three way decision improves the identification of autism patients from multisite f-MRI data. *Brain Sciences*.

[77] Grana M., Silva M. (2021). Impact of machine learning pipeline choices in autism prediction from functional connectivity data. *International Journal of Neural Systems*.

[78] Spera G., Retico A., Bosco P. (2019). Evaluation of altered functional connections in male children with autism spectrum disorders on multiple-site data optimized with machine learning. *Frontiers in Psychiatry*.

[79] Reiter M. A., Jahedi A., Fredo A. R. J., Fishman I., Bailey B., Muller R. A. (2021). Performance of machine learning classification models of autism using resting state fmri is contingent on sample heterogeneity. *Neural Computing & Applications*.

[80] Chaitra N., Vijaya P. A., Deshpande G. (2020). Diagnostic prediction of autism spectrum disorder using complex network measures in a machine learning framework. *Biomedical Signal Processing and Control*.

[81] Brahim A., Farrugia N. (2020). Graph fourier transform of fmri temporal signals based on an averaged structural connectome for the classification of neuroimaging. *Artificial Intelligence in Medicine*.

[82] Sun J. W., Fan R., Wang Q., Wang Q. Q., Jia X. Z., Ma H. B. (2021b). Identify abnormal functional connectivity of resting state networks in autism spectrum disorder and apply to machine learning based classification. *Brain Research*.

[83] Kazeminejad A., Sotero R. C. (2020). The importance of anti correlations in graph theory based classification of autism spectrum disorder. *Frontiers in Neuroscience*.

[84] Jamwal I., Malhotra D., Mengi M. (2022). Autism spectrum disorder detection using asd_sfmri. *Computer Vision and Robotics*.

[85] Traut N., Heuer K., Lemaître G. (2022). Insights from an autism imaging biomarker challenge: promises and threats to biomarker discovery. *NeuroImage*.

[86] Dadi K., Rahim M., Abraham A. (2019). Benchmarking functional connectome-based predictive models for resting-state f-MRI. *NeuroImage*.

[87] Abraham A., Milham M. P., Di Martino A. (2017). Deriving reproducible biomarkers from multi-site resting-state data: an Autism-based example. *NeuroImage*.

[88] Gogoi D. K., Talukdar J., Bhattacharyya D. K., Singh T. P. (2023). A deep learning approach to classify autism spectrum disorder using MRI images. *Preprints*.

[89] Han T., Gong X., Feng F., Zhang J., Sun Z., Zhang Y. (2023). Privacy-preserving multi-source domain adaptation for medical data. *IEEE Journal of Biomedical and Health Informatics*.

[90] Manikantan K., Jaganathan S. (2023). A model for diagnosing autism patients using spatial and statistical measures using rs-fMRI and sMRI by adopting graphical neural networks. *Diagnostics*.

[91] Deng X., Zhang J., Liu R., Liu K. (2022). Classifying ASD based on time-series fMRI using spatial-temporal transformer. *Computers in Biology and Medicine*.

[92] Dhinagar N. J., Santhalingam V., Lawrence K. E., Laltoo E., Thompson P. M. (2023). Few-shot classification of autism spectrum disorder using site-agnostic meta-learning and brain mri. https://arxiv.org/abs/2303.08224.

[93] Artiles O., Al Masry Z., Saeed F. (2023). Confounding effects on the performance of machine learning analysis of static functional connectivity computed from rs-fMRI multi-site data. *Neuroinformatics*.

[94] Quiang N., Gao J., Dong Q. (2023). A deep learning method for autism spectrum disorder identification based on interactions of hierarchical brain networks. *Behavioural Brain Research*.

[95] Jönemo J., Abramian D., Eklund A. (2023). Evaluation of augmentation methods in classifying autism spectrum disorders from fmri data with 3d convolutional neural networks. *Diagnostics (Basel)*.

[96] Kunda M., Zhou S., Gong G., Lu H. (2023). Improving multi-site autism classification via site-dependence minimization and second-order functional connectivity. *IEEE Transactions on Medical Imaging*.

[97] Libero L. E., DeRamus T. P., Lahti A. C., Deshpande G., Kana R. K. (2015). Multimodal neuroimaging based classification of autism spectrum disorder using anatomical, neurochemical, and white matter correlates. *Cortex*.

